# Association of Increasing the Minimum Wage in the US With Experiences of Maternal Stressful Life Events

**DOI:** 10.1001/jamanetworkopen.2023.24018

**Published:** 2023-07-18

**Authors:** Slawa Rokicki, Nancy E. Reichman, Mark E. McGovern

**Affiliations:** 1 Department of Health Behavior, Society and Policy, Rutgers School of Public Health, Piscataway, New Jersey; 2 Department of Pediatrics, Rutgers Robert Wood Johnson Medical School, New Brunswick, New Jersey; 3 Child Health Institute of New Jersey, Rutgers Robert Wood Johnson Medical School, New Brunswick, New Jersey; 4 Department of Economics, Princeton University, Princeton, New Jersey

## Abstract

**Importance:**

Exposure to stressful life events (SLEs) before and during pregnancy is associated with adverse health for pregnant people and their children. Minimum wage policies have the potential to reduce exposure to SLEs among socioeconomically disadvantaged pregnant people.

**Objective:**

To examine the association of increasing the minimum wage with experience of maternal SLEs.

**Design, Setting, and Participants:**

This repeated cross-sectional study included 199 308 individuals who gave birth between January 1, 2004, and December 31, 2015, in 39 states that participated in at least 2 years of the Pregnancy Risk Assessment Monitoring Survey between 2004 and 2015. Statistical analysis was performed from September 1, 2022, to January 6, 2023.

**Exposure:**

The mean minimum wage in the 2 years prior to the month and year of delivery in an individual’s state of residence.

**Main Outcomes and Measures:**

The main outcomes were number of financial, partner-related, traumatic, and total SLEs in the 12 months before delivery. Individual-level covariates included age, race and ethnicity, marital status, parity, educational level, and birth month. State-level covariates included unemployment, gross state product, uninsurance, poverty, state income supports, political affiliation of governor, and Medicaid eligibility levels. A 2-way fixed-effects analysis was conducted, adjusting for individual and state-level covariates and state-specific time trends.

**Results:**

Of the 199 308 women (mean [SD] age at delivery, 25.7 [6.1] years) in the study, 1.4% were American Indian or Alaska Native, 2.5% were Asian or Pacific Islander, 27.2% were Hispanic, 17.6% were non-Hispanic Black, and 48.8% were non-Hispanic White. A $1 increase in the minimum wage was associated with a reduction in total SLEs (−0.060; 95% CI, −0.095 to −0.024), financial SLEs (−0.032; 95% CI, −0.056 to −0.007), and partner-related SLEs (−0.019; 95% CI, −0.036 to −0.003). When stratifying by race and ethnicity, minimum wage increases were associated with larger reductions in total SLEs for Hispanic women (−0.125; 95% CI, −0.242 to −0.009).

**Conclusions and Relevance:**

In this repeated cross-sectional study of women with a high school education or less across 39 states, an increase in the state-level minimum wage was associated with reductions in experiences of maternal SLEs. Findings support the potential of increasing the minimum wage as a policy for improving maternal well-being among socioeconomically disadvantaged pregnant people. These findings have relevance for current policy debates regarding the minimum wage as a tool for improving population health.

## Key Points

**Question:**

Is there an association between increasing the minimum wage in the US and experiences of maternal stressful life events among socioeconomically disadvantaged pregnant people?

**Findings:**

In this cross-sectional study including 199 308 women across 39 states, a $1 increase in the state-level minimum wage was associated with significant reductions in experiences of financial, partner-related, and total stressful life events. The largest reductions were observed for Hispanic women.

**Meaning:**

The findings suggest that there is potential for minimum wage policies to improve maternal well-being among socioeconomically disadvantaged pregnant people.

## Teaser

This cross-sectional study examines the association of increasing the minimum wage in the US with the experience of maternal stressful life events among women with a high school education or less.

## Introduction

Exposure to stressful life events (SLEs) before and during pregnancy is associated with serious adverse health for pregnant people and their children. A large amount of the literature shows an association of exposure to maternal SLEs, defined as experiences or events that cause severe strain, such as job loss or divorce, with increased risk of preterm birth, low birth weight, small size for gestational age, gestational hypertension, and peripartum depression.^1,2,3,4,5,6^

As social determinants of health, SLEs play a central role in the maternal health crisis in the US.^7,8,9^ Non-Hispanic Black (hereafter, *Black*) and American Indian or Alaska Native women have 2 to 3 times the risk of pregnancy-related mortality as non-Hispanic White (hereafter, *White*) women and elevated risks of adverse birth outcomes.^10,11^ American Indian or Alaska Native, Black, and Hispanic groups experience disproportionately high rates of poverty,^12^ which increases exposure to SLEs associated with economic hardship and is associated with greater family conflict and disruption of relationships.^13^ Approximately 60% of women living in poverty or near poverty experience at least 1 SLE during or just before pregnancy; those at highest risk are unmarried, have lower income and educational levels, and are younger than 25 years.^12,14^ American Indian or Alaska Native, Black, and Hispanic women have significantly higher prevalences of financial and partner-related SLEs compared with White women.^14,15^ Differences in exposure to SLEs may therefore be an important factor in the persistent racial and ethnic disparities in maternal health and birth outcomes.^16^

From a population health perspective, addressing upstream structural conditions through public policy may be most effective at reducing maternal health inequities.^7,8,17^ One public policy that has been attracting increasing attention within the public health community is increasing the minimum wage.^18,19^ Although minimum wage policies have been the subject of empirical study within the economics literature, emerging evidence on their health effects has led to increasing interest in their wider health and social effects.^18,19^

Evidence on the association of the minimum wage with public health is needed because of the frequency with which state-level minimum wage policies are changing. Although the federal minimum wage, at $7.25, has not changed since 2009, many states have implemented their own laws. In 2022, state minimum wages ranged widely, from $7.25 to $16.10. Estimates indicate that increasing the minimum wage from $7.25 to $12.00 would lift 6.6 million people out of poverty, with negligible effects on unemployment.^19^ Those earning the minimum wage are disproportionately likely to be female and members of racial and ethnic minority groups; thus, there is a large potential for minimum wage laws to enhance maternal well-being and reduce racial and ethnic health inequities.^19,20^

Two studies found that increasing the minimum wage was associated with a reduced risk of low birth weight and neonatal death.^21,22^ Neither changes in prenatal care use nor smoking behavior appeared to be mechanisms for the observed reductions, leaving open the question of the channels through which minimum wage is associated with birth outcomes.^22^ Given the strong association of maternal stress with maternal and infant health, the stress pathway has the potential to account for a significant share of the association. Recent work has found that, among the general population, a higher minimum wage is associated with reduced suicide rates and improved mental health, likely as a result of decreased financial stress.^23,24,25^ However, to our knowledge, there is currently little evidence on the association of the minimum wage with experiences of stress or SLEs, particularly among pregnant people.^26,27^ Moreover, few studies have examined the differential effects of the minimum wage by race and ethnicity or other population characteristics.^28^

Using population-based state-level data from 39 states over 12 years, we investigated the association between state-level minimum wage and maternal SLEs. We also conducted stratified analyses by race and ethnicity to examine the potential of minimum wages to improve maternal health equity. We hypothesized that higher minimum wages would be associated with reductions in the number of maternal SLEs, with the largest associations for women from racial and ethnic minority groups.

## Methods

### Data and Analysis Sample

We used data from the Centers for Disease Control and Prevention (CDC) Pregnancy Risk Assessment Monitoring System (PRAMS) between January 1, 2004, and December 31, 2015.^29^ For each participating state and year, birth certificate records are used to select a representative sample of women who delivered a live-born infant. We use the term *women* for this sample to maintain consistency with PRAMS; however, no gender identity questions were asked. The data were deidentified, and this study was deemed exempt from review by the Rutgers University institutional review board. Informed consent was not required because it was implied by completion of the survey by the respondent. This study followed the Strengthening the Reporting of Observational Studies in Epidemiology (STROBE) reporting guideline for cross-sectional studies.

States must meet the response rate threshold for PRAMS data to be available for researchers, and this rate has changed over time: 70% until 2006, 65% for 2007 to 2011, 60% for 2012 to 2014, and 55% for 2015. In addition, the number of states participating in PRAMS has changed over time. As a result, the composition of states changes across years. Consistent with prior research,^27,30^ in our main analysis, we included all states with at least 2 years of data over the study period (39 states comprising 459 037 individuals) (eFigure in Supplement 1). We excluded women who reported having more than a high school education, because changes in the minimum wage are not likely to be associated with outcomes for this group (n = 250 650), and those with missing education (n = 6042).^22,28^ We also excluded those with missing data for all SLEs (n = 3037). The final analysis sample size was 199 308. Yearly data on state minimum wages and other state-level policies were obtained from the University of Kentucky Center for Poverty Research, and Medicaid eligibility levels were obtained from the Kaiser Family Foundation.

### Outcome

Our outcome of interest was maternal SLEs in the year before delivery, derived from a subset of the Modified Life Events Inventory.^31^ Across the study period, 12 items were consistently reported and 9 were specific to the experience of the respondent. Following CDC guidelines and a large literature on the use of SLEs in PRAMS data,^14,32,33^ we categorized those 9 SLEs into 3 domains: (1) financial (moved to a new address, respondent lost job, partner lost job, or unable to pay bills), (2) partner-related (separated or divorced, argued more than usual with husband or partner, or husband or partner said they did not want pregnancy), and (3) traumatic (was homeless or partner or respondent went to jail). We created outcome variables for the total number of stressors, both overall and within each domain.

### Exposure

Because outcomes were measured in the 12 months before delivery, we defined our exposure to be the mean of the continuous state-level minimum wage in the 2 years prior to the month and year of delivery. For states with no minimum wage law and states with laws below the federal minimum, the effective minimum wage was defined as the higher of the state and federal minimum wage in each state. For the main analysis, we transformed nominal minimum wages into 2020 dollars using the annual mean Consumer Price Index. In sensitivity analyses, we report results for other ways of defining the exposure, including using the nominal minimum wage and using the minimum wage in the calendar year before the delivery year.

### Covariates

We adjusted models for individual characteristics including mother’s age, race and ethnicity, marital status, parity, educational level, and month of child’s birth. To classify race and ethnicity, we used variables provided in PRAMS that are derived from the birth certificate and are self-reported. The final categories included American Indian or Alaska Native, Asian or Pacific Islander, Black, Hispanic, White, and other (which included subcategories of other race, mixed race, and missing race). We included race and ethnicity variables because individuals from racial and ethnic minority groups are disproportionately affected by minimum wages and because of the large and systemic racial disparities in maternal health outcomes.^16,20^ Individuals with missing covariate information were coded using a separate missing category indicator or as part of a larger category.

We also adjusted for state-level, time-varying characteristics that were lagged by 2 years from year of delivery, including unemployment rate, gross state product, percentage of children uninsured, poverty rate, state earned income tax credit rate as percentage of federal rate, combined monthly maximum for Temporary Assistance for Needy Families (TANF) and Supplemental Nutrition Assistance Program (SNAP) benefits for a 2-person family, state governor’s political affiliation, and Medicaid eligibility level as percentage of the federal poverty limit for pregnant women and parents.

### Statistical Analysis

Data analysis was performed from September 1, 2022, to January 6, 2023. We estimated the association of the state-level minimum wage with outcomes using a 2-way fixed-effects (TWFE) approach.^34^ We used linear regression models for our primary analysis; we also compared results with Poisson models. In addition to the exposure variable and individual covariates, model 1 included state and year indicators. These fixed effects account for time-invariant differences between states and changes over time that are common across states. The primary threat to validity is if unmeasured, time-varying, state-specific factors are correlated with both changes in the minimum wage and outcomes. To address this possibility, we additionally controlled for a range of state-level, time-varying characteristics (model 2) and included state-specific linear time trends to control for residual confounding taking the form of secular changes at the state level (model 3).^22^ Other requirements for TWFE models to yield consistent estimates are that confounders are linearly additive and that there is homogeneity in treatment effects.^34,35^ To account for within-state autocorrelation, SEs were clustered at the state level.^36^ We also stratified models by race and ethnicity, age, and marital status. All analyses were weighted using survey weights provided by the CDC to account for the sampling design. We used 2-sided statistical tests and 95% CIs, with *P* < .05 considered significant. We estimated the expected percentage change in SLEs for a proposed change in the federal minimum wage by calculating the difference in the adjusted linear estimates for $7.25 and $12.00 (using model 3), divided by the outcome mean. Analysis was conducted using Stata, version 17 (StataCorp LP).

We conducted several sensitivity analyses to assess the robustness of the results. To reduce imbalance in the data from states moving in and out of participating in PRAMS, we restricted the sample to states with fewer than 3 years of missing data over the study period (24 states). We also expanded the study period to 2019 for the states that included SLEs as part of their state’s questionnaire (these questions were no longer mandatory after 2015). Next, we used alternative measures of the exposure, as already described. Finally, we adjusted for within-state correlation using the wild cluster bootstrap in Stata.^36^

We also conducted falsification tests to assess the validity of our analysis. We reestimated models among the sample of women with more than a high school education because minimum wage changes are unlikely to be associated with outcomes for this group.^22^ We also examined whether there were associations with minimum wage changes that occurred 1, 2, or 4 years after delivery because future changes should not be associated with outcomes in the year before delivery.

## Results

The final analysis sample size was 199 308 individuals (mean [SD] age at delivery, 25.7 [6.1] years) with a high school education or less who gave birth in the past year (Table 1). In this population, 1.4% were American Indian or Alaska Native, 2.5% were Asian or Pacific Islander, 17.6% were Black, 27.2% were Hispanic, and 48.8% were White. A total of 38.0% of participants did not complete high school, 59.3% were not married, and 38.8% were nulliparous. A total of 67.8% of participants reported having experienced at least 1 SLE.

**Table 1.** Descriptive Characteristics of Analysis Sample of Women With High School Education or Less, 2004-2015

Characteristic	No. (%) (N = 199 308)^a^
Age at delivery, y	
<20	38 051 (18.1)
20-29	114 498 (58.5)
30-39	42 850 (21.5)
≥40	3909 (1.8)
Race and ethnicity	
American Indian or Alaska Native	10 436 (1.4)
Asian or Pacific Islander	9688 (2.5)
Black	39 028 (17.6)
Hispanic	45 641 (27.2)
White	88 038 (48.8)
Other^b^	6477 (2.4)
Educational level	
Less than high school	73 969 (38.0)
High school diploma	125 339 (62.0)
Marital status	
Not married or missing	119 201 (59.3)
Married	80 107 (40.7)
Nulliparous	
No	115 669 (59.6)
Yes	80 452 (38.8)
Missing	3187 (1.6)
SLEs	
Experienced any SLEs	137 752 (67.8)
Total No. of SLEs, median (range)	1 (0-9)
Total No. of partner-related SLEs, median (range)	0 (0-3)
Total No. of financial SLEs, median (range)	1 (0-4)
Total No. of traumatic SLEs, median (range)	0 (0-2)

Abbreviation: SLEs, stressful life events.

^a^ Estimates are unweighted sample sizes and survey-weighted percentages.

^b^ Other race and ethnicity category includes the subcategories of other race, mixed race, and missing race.

**Figure 1.**
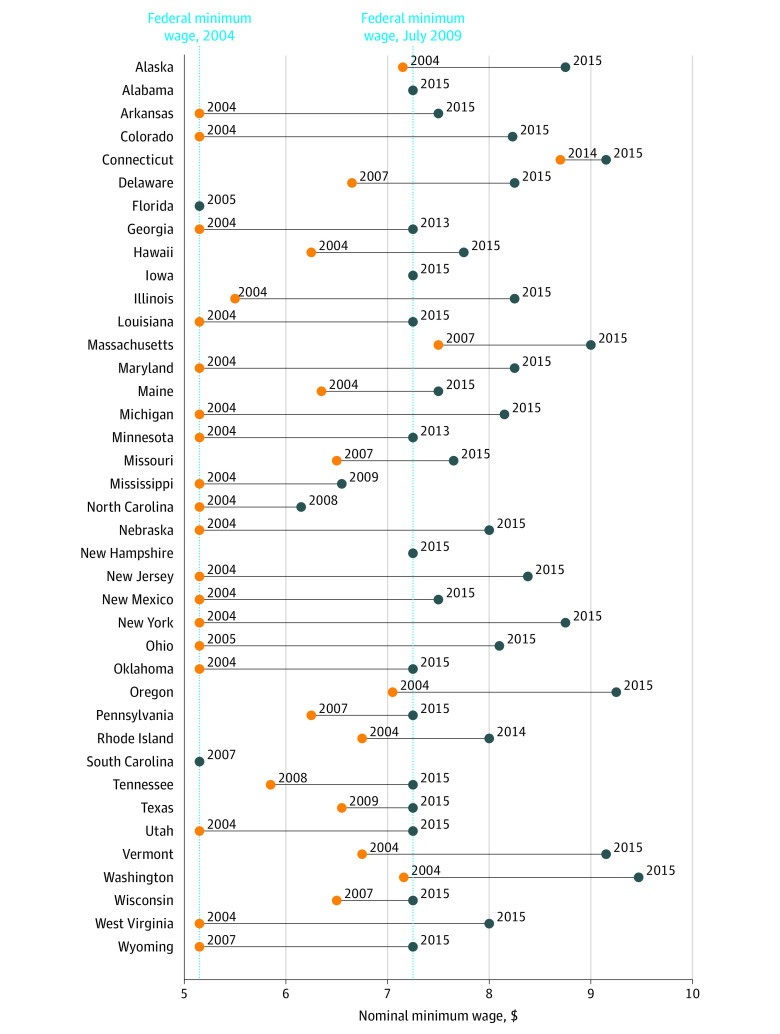
Change in Minimum Wage by Year in the 39 States in the Study Sample, 2004-2015 Five states (Florida [2004-2005], Alabama [2014-2015], South Carolina [2004-2007], Iowa [2013-2015], and New Hampshire [2013-2015]) did not change their minimum wage over the observed years in the sample data. The federal minimum wage changed in July 2009, while data on state minimum wages provide the minimum wage on January 1 of the year.

Figure 1 shows the minimum wage in the first and last year of available data for each of the 39 states included in the sample. There was substantial variation in minimum wages both across and within states. In 2004, the lowest and highest minimum wages were $5.15 and $7.16. In 2015, the lowest and highest minimum wages were $7.25 and $9.47.

Table 2 shows the results of the TWFE analysis. In model 3, a $1 increase in the minimum wage was associated with a reduction in total SLEs of 0.060 (95% CI, −0.095 to −0.024). This estimate translates into an 18% (95% CI, 8%-28%) relative decrease in total SLEs for women in affected states when increasing the federal minimum wage from $7.25 to $12.00.

**Table 2.** Two-Way Fixed Effect Estimates of a $1 Increase in the Minimum Wage on SLEs in the 12 Months Before Delivery, 2004-2015

Outcomes	Model 1	Model 2	Model 3	No.	Sample, mean (SE)	Change from $7.25 to $12.00 relative to mean, %^a^
Total SLEs	−0.023 (−0.057 to 0.010)	−0.040 (−0.079 to −0.001)^b^	−0.060 (−0.095 to −0.024)^c^	199 308	1.57 (0.007)	−18 (−28 to −8)
Partner-related SLEs	−0.014 (−0.025 to −0.002)^b^	−0.020 (−0.035 to −0.006)^c^	−0.019 (−0.036 to −0.003)^b^	199 048	0.51 (0.003)	−17 (−33 to −3)
Financial SLEs	−0.005 (−0.028 to 0.018)	−0.013 (−0.039 to 0.012)	−0.032 (−0.056 to −0.007)^b^	199 181	0.94 (0.004)	−16 (−28 to −4)
Traumatic SLEs	−0.005 (−0.016 to 0.007)	−0.006 (−0.018 to 0.005)	−0.009 (−0.021 to 0.004)	198 879	0.13 (0.001)	−33 (−77 to 15)
Model specifications						
Individual controls	Yes	Yes	Yes	NA	NA	NA
State and time fixed effects	Yes	Yes	Yes	NA	NA	NA
State time-varying controls	No	Yes	Yes	NA	NA	NA
State linear time trends	No	No	Yes	NA	NA	NA

Abbreviations: NA, not applicable; SLEs, stressful life events.

^a^ Uses model 3 estimate. Individual controls include maternal age, race and ethnicity, marital status, parity, educational level, and month of child’s birth. State time-varying controls include unemployment rate, gross state product, percentage of children uninsured, poverty rate, state earned income tax credit rate as a percentage of the federal rate, combined monthly maximum for Temporary Assistance for Needy Families and Supplemental Nutrition Assistance Program benefits for a 2-person family, whether the governor was affiliated with the Democratic party, and Medicaid eligibility level as a percentage of the federal poverty limit for pregnant women and for parents. The 95% CIs reflect clustered SEs.

^b^ *P* < .05.

^c^ *P* < .01.

In model 3, financial-related SLEs had the largest decreases associated with increases in the minimum wage (−0.032; 95% CI, −0.056 to −0.007) (Table 2). Partner-related SLEs were also negatively associated with the minimum wage (−0.019; 95% CI, −0.036 to −0.003). Traumatic SLEs were not associated with the minimum wage (–0.009; 95% CI, –0.021 to 0.004). Full specification results are shown in eTable 1 in Supplement 1. Results for individual SLEs are shown in eTable 2 in Supplement 1; significant associations were observed for the following outcomes: not being able to pay bills, moving to a new address, husband or partner not wanting the pregnancy, and husband or partner going to jail. Alternative estimates using Poisson models are shown in eTable 3 in Supplement 1 and are consistent with the results of the linear models.

**Figure 2.**
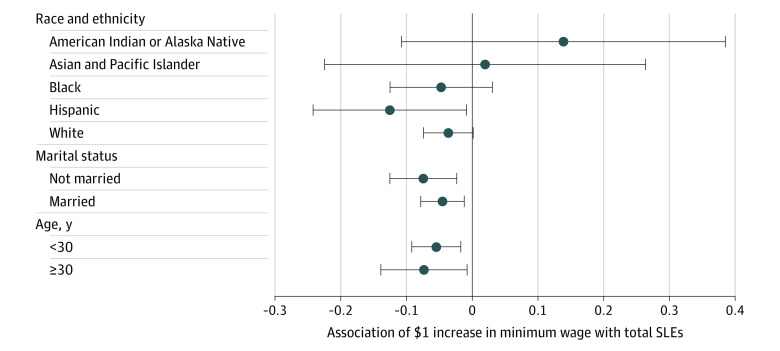
Heterogeneous Estimates of the Association of a $1 Increase in the Minimum Wage With the Total Number of Stressful Life Events (SLEs), by Race and Ethnicity, Age, and Marital Status, 2004-2015 Results of stratified models are shown, with each model adjusted for individual covariates, state time-varying controls, state and year fixed effects, and state linear time trends.

Figure 2 shows adjusted estimates of the association of a $1 increase in the minimum wage with total SLEs in models stratified by race and ethnicity, marital status, and age. Minimum wage increases were associated with larger reductions in total SLEs for Hispanic women based on the magnitude of the coefficient (−0.125; 95% CI, −0.242 to −0.009); however, due to smaller sample sizes, 95% CIs were wide and overlapping across groups (eTable 4 in Supplement 1).

Results were robust to alternative model specifications (eTable 5 in Supplement 1). As expected, we found no association between minimum wages and total SLEs among the sample of college-educated women or for future exposures, which strengthens confidence in the validity of our analytic approach.

## Discussion

We found evidence that a $1 increase in the state-level minimum wage was associated with reductions in the experience of maternal SLEs, including total, partner-related, and financial SLEs, among women with a high school education or less. Considering the population of affected individuals and a proposed increase in the federal minimum wage in line with that being considered in Congress, the overall consequences of increasing the wages of those at the lower end of the income distribution appear substantial. We estimate that an increase in the minimum wage from $7.25 to $12.00 could translate to an 18% (95% CI, 8%-28%) decrease in total SLEs among socioeconomically disadvantaged pregnant people in affected states.

Results for individual SLEs were consistent with the hypothesis that increases in minimum wages may reduce stress associated with financial insecurity because we found significant negative associations between the minimum wage and inability to pay bills and moving to a new address. There was also a significant negative association with the partner not wanting the pregnancy; desire for children is closely tied with economic security.^37^ Research examining the association of the minimum wage with food insecurity and housing instability among pregnant people would help to further illuminate pathways between minimum wages, financial insecurity, and stress. Future research should also aim to quantify how these economic mechanisms translate into associations with health for both mothers and children.

In stratified analyses, minimum wage increases were associated with larger reductions in total SLEs for Hispanic women, which was consistent with our hypothesis given that this population of workers is disproportionately likely to be earning the minimum wage. However, for Black women, the estimate of the association between minimum wages and SLEs, while slightly larger in magnitude than that for White women, was not statistically significant at the .05 level. The estimate for American Indian or Alaska Native women was imprecise due to the small sample size for this group. Because of the width of the 95% CIs, caution is warranted in comparing results in these stratified analyses. Nevertheless, if confirmed in additional data, these results would have implications for the potential of minimum wage policies to address maternal health inequities. Overall, Hispanic women have similar rates of adverse birth and pregnancy outcomes as White women; however, rates are highly heterogenous with regard to nativity and country of origin, so a higher minimum wage may reduce health inequities within Hispanic subgroups.^38^ Emerging research shows that American Indian or Alaska Native and Black women experience chronic racism-related stress in conjunction with poverty-related stress, which is associated with higher levels of SLEs regardless of income.^11,39^ Research examining multilevel interventions addressing structural racism and income inequality is needed to better understand potentially heterogeneous effects within racial and ethnic groups.

Our findings contribute to the literature on the association of economic policies with maternal health. Our results are consistent with recent evaluations of the association of minimum wage increases with health outcomes, including reductions in adverse birth outcomes, improvements in mental health, reductions in suicides, and decreases in divorce rates.^22,23,24,25,27,40^ They also align with evidence for financial support programs, such as the Earned Income Tax Credit,^41^ TANF,^42,43^ and SNAP,^44^ in improving maternal health and reducing parental stress.

### Strengths and Limitations

This study had several strengths. PRAMS data follow a standardized data collection method that results in consistent measures of SLEs across states and time over 12 years, allowing for evaluation of state-level policy changes.^29^ The study period spans important federal and state changes in minimum wages, allowing us to leverage variation across states and time and account for a broad set of unobserved confounders. Numerous robustness checks and falsification tests strengthen confidence in the validity of the analytic strategy.

This study also had some limitations. We adopted a TWFE approach with continuous treatment in which we assume the associations of the minimum wage with outcomes are linear; research has found bias in this context in the presence of treatment effect heterogeneity or nonlinearities in dose response.^34,45,46^ Because PRAMS data are imbalanced, with states lacking consistent preperiods and postperiods, other more balanced data sources are better suited to the implementation of methodological alternatives, such as event study analysis or continuous difference-in-differences analysis with alternative parallel trends assumptions. Our outcome was a summary measure of 9 SLEs, which may not adequately capture all types of adverse events. Because the focus is on specific events, this summary measure also does not capture chronic stress.

## Conclusions

A growing body of literature finds that the unequal distribution of stressors in society is a primary mechanism associated with health inequities in socially and economically disadvantaged communities.^47^ There is a strong and established association between social determinants and stress and between stress and maternal and child health.^1,14,47,48^ Identifying public policies that can reduce exposure to stressful events among pregnant people may have significant downstream effects on birth outcomes, maternal health, and child health and development. Moreover, considering the large and persistent racial and ethnic disparities in maternal and child health, proequity policies that may reduce health disparities are of particular public policy relevance.^49,50^ The results of this repeated cross-sectional study provide evidence that increasing the minimum wage, a policy that would disproportionately benefit racial and ethnic minority communities, is associated with a reduction in experiences of SLEs among pregnant people.

## Notes

Supplement 1. **eFigure.** Sample Construction **eTable 1.** Two-Way Fixed Effect Estimates (Full Specification Results) of a $1 Increase in the Minimum Wage on Stressful Life Events in the 12 Months Before Delivery, 2004-2015 **eTable 2.** Two-Way Fixed Effect Estimates of $1 Increase in Minimum Wage on Individual Stressful Events in the 12 Months Before Delivery, 2004-2015 **eTable 3.** Incidence Rate Ratio Estimates Using Poisson Regression Models of $1 Increase in Minimum Wage on Stressful Life Events in the 12 Months Before Delivery, 2004-2015 **eTable 4.** Two-Way Fixed Effect Estimates of $1 Increase in Minimum Wage in the 12 Months Before Delivery on Total Stressful Life Events, Stratified by Characteristics, 2004-2015 **eTable 5.** Results of Robustness Checks and Falsification Tests

Supplement 2.
Data Sharing Statement
