# Supplementary material for: Association of Increasing the Minimum Wage in the US With Experiences of Maternal Stressful Life Events
Source: JAMA Netw Open. 2023 Jul 18;6(7):e2324018. doi: 10.1001/jamanetworkopen.2023.24018 (PMC10354676; doi:10.1001/jamanetworkopen.2023.24018)
Supplement: Supplement 1. — eFigure. Sample Construction eTable 1. Two-Way Fixed Effect Estimates (Full Specification Results) of a $1 Increase in the Minimum Wage on Stressful Life Events in the 12 Months Before Delivery, 2004-2015 eTable 2. Two-Way Fixed Effect Estimates of $1 Increase in Minimum Wage on Individual Stressful Events in the 12 Months Before Delivery, 2004-2015 eTable 3. Incidence Rate Ratio Estimates Using Poisson Regression Models of $1 Increase in Minimum Wage on Stressful Life Events in the 12 Months Before Delivery, 2004-2015 eTable 4. Two-Way Fixed Effect Estimates of $1 Increase in Minimum Wage in the 12 Months Before Delivery on Total Stressful Life Events, Stratified by Characteristics, 2004-2015 eTable 5. Results of Robustness Checks and Falsification Tests [file jamanetwopen-e2324018-s001.pdf]

Supplementary Online Content

Rokicki S, Reichman NE, McGovern ME. Association of increasing the minimum wage in the US with experiences of maternal stressful life events. *JAMA Netw Open.* 2023;6(7):e2324018. doi:10.1001/jamanetworkopen.2023.24018

eFigure. Sample Construction

eTable 1. Two-Way Fixed Effect Estimates (Full Specification Results) of a $1 Increase in the Minimum Wage on Stressful Life Events in the 12 Months Before Delivery, 2004-2015

eTable 2. Two-Way Fixed Effect Estimates of $1 Increase in Minimum Wage on Individual Stressful Events in the 12 Months Before Delivery, 2004-2015

eTable 3. Incidence Rate Ratio Estimates Using Poisson Regression Models of $1 Increase in Minimum Wage on Stressful Life Events in the 12 Months Before Delivery, 2004-2015

eTable 4. Two-Way Fixed Effect Estimates of $1 Increase in Minimum Wage in the 12 Months Before Delivery on Total Stressful Life Events, Stratified by Characteristics, 2004-2015

eTable 5. Results of Robustness Checks and Falsification Tests

This supplementary material has been provided by the authors to give readers additional information about their work.

© 2023 Rokicki S et al. *JAMA Network Open.*

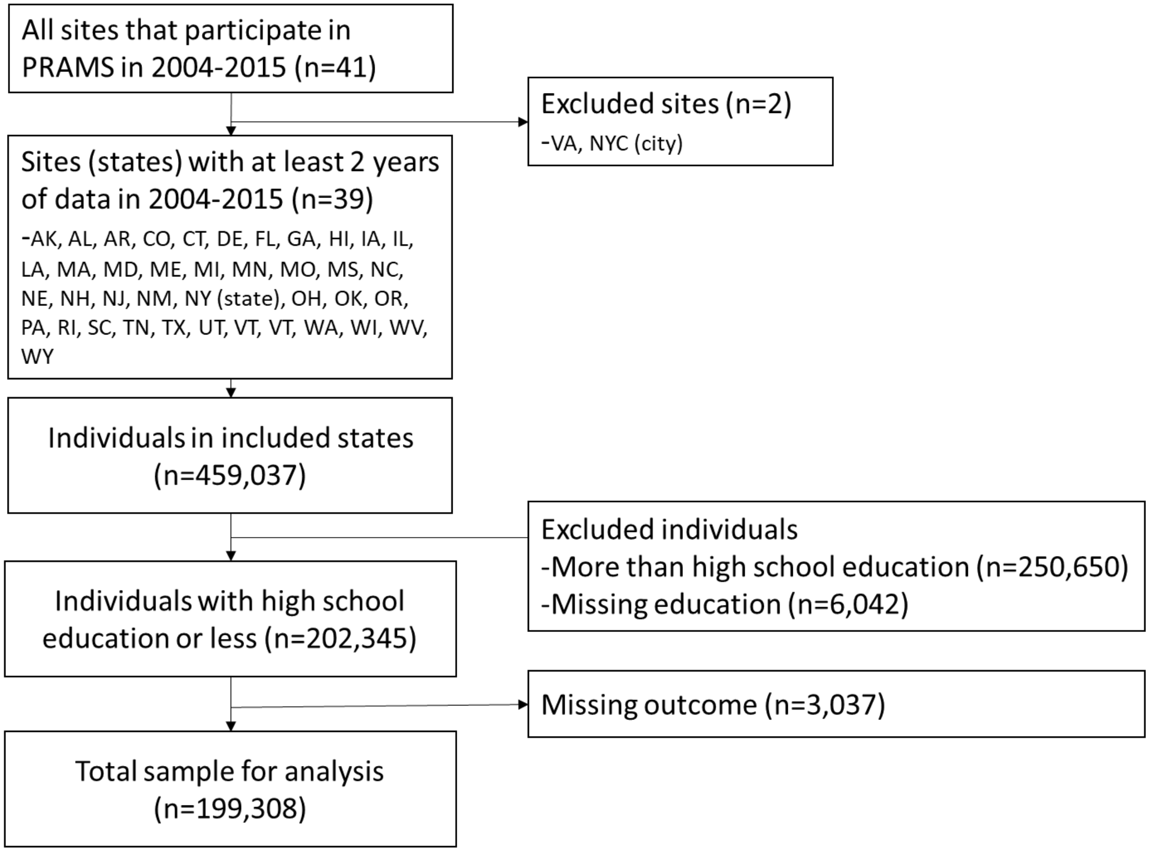

eFigure. Sample Construction

© 2023 Rokicki S et al. *JAMA Network Open.*

eTable 1. Two-Way Fixed Effect Estimates (Full Specification Results) of a $1 Increase in the Minimum Wage on Stressful Life Events in the 12 Months Before Delivery, 2004-2015

VARIABLES	(1) Total number of stressors, Indiv controls	(2) Total number of stressors, State controls	(3) Total number of stressors, State time trends	(4) Total partner stress, Indiv controls	(5) Total partner stress, State controls	(6) Total partner stress, State time trends	(7) Total financial stress, Indiv controls	(8) Total financial stress, State controls	(9) Total financial stress, State time trends	(10) Total traumatic stress, Indiv controls	(11) Total traumatic stress, State controls	(12) Total traumatic stress, State time trends
Minimum wage	-0.023 (-0.057 to 0.010)	-0.040** (-0.079 to -0.000)	-0.060*** (-0.095 to -0.024)	-0.014** (-0.025 to -0.002)	-0.020*** (-0.035 to -0.006)	-0.019** (-0.036 to -0.003)	-0.005 (-0.028 to 0.018)	-0.013 (-0.039 to 0.012)	-0.032** (-0.056 to -0.007)	-0.005 (-0.016 to 0.007)	-0.006 (-0.018 to 0.005)	-0.009 (-0.021 to 0.004)
Age at delivery = 2, 20-29	0.117*** (0.091 to 0.144)	0.117*** (0.091 to 0.143)	0.118*** (0.092 to 0.144)	-0.086*** (-0.101 to -0.072)	-0.086*** (-0.101 to -0.072)	-0.086*** (-0.100 to -0.071)	0.199*** (0.178 to 0.219)	0.199*** (0.178 to 0.219)	0.199*** (0.178 to 0.219)	0.005 (-0.004 to 0.015)	0.005 (-0.004 to 0.015)	0.005 (-0.005 to 0.015)
Age at delivery = 3, 30-39	-0.202*** (-0.250 to -0.153)	-0.200*** (-0.249 to -0.152)	-0.200*** (-0.248 to -0.151)	-0.163*** (-0.181 to -0.145)	-0.163*** (-0.181 to -0.144)	-0.163*** (-0.181 to -0.144)	-0.016 (-0.049 to 0.017)	-0.016 (-0.049 to 0.018)	-0.015 (-0.048 to 0.018)	-0.022*** (-0.033 to -0.010)	-0.022*** (-0.033 to -0.010)	-0.021*** (-0.033 to -0.010)
Age at delivery = 4, 40+	-0.214*** (-0.324 to -0.104)	-0.211*** (-0.321 to -0.102)	-0.211*** (-0.321 to -0.102)	-0.143*** (-0.183 to -0.103)	-0.142*** (-0.182 to -0.102)	-0.142*** (-0.183 to -0.102)	-0.037 (-0.113 to 0.039)	-0.035 (-0.111 to 0.040)	-0.036 (-0.111 to 0.039)	-0.033*** (-0.051 to -0.014)	-0.032*** (-0.051 to -0.014)	-0.032*** (-0.050 to -0.014)
Race = 2, Black	-0.046* (-0.102 to 0.009)	-0.046 (-0.101 to 0.009)	-0.046* (-0.100 to 0.009)	0.050*** (0.025 to 0.075)	0.050*** (0.026 to 0.075)	0.050*** (0.026 to 0.075)	-0.124*** (-0.153 to -0.095)	-0.124*** (-0.153 to -0.094)	-0.123*** (-0.153 to -0.094)	0.028*** (0.018 to 0.039)	0.029*** (0.018 to 0.039)	0.028*** (0.018 to 0.039)
Race = 3, Asian/PI	-0.351*** (-0.422 to -0.280)	-0.353*** (-0.423 to -0.282)	-0.359*** (-0.430 to -0.289)	-0.099*** (-0.128 to -0.069)	-0.099*** (-0.128 to -0.070)	-0.102*** (-0.131 to -0.072)	-0.205*** (-0.253 to -0.156)	-0.206*** (-0.255 to -0.157)	-0.209*** (-0.258 to -0.160)	-0.047*** (-0.061 to -0.032)	-0.047*** (-0.060 to -0.033)	-0.048*** (-0.061 to -0.034)
Race = 4, AI/AN	-0.055 (-0.183 to 0.073)	-0.055 (-0.184 to 0.074)	-0.056 (-0.185 to 0.073)	0.008 (-0.042 to 0.058)	0.008 (-0.042 to 0.058)	0.008 (-0.042 to 0.058)	-0.116*** (-0.184 to -0.047)	-0.116*** (-0.184 to -0.047)	-0.117*** (-0.186 to -0.048)	0.053*** (0.014 to 0.092)	0.053*** (0.014 to 0.092)	0.053*** (0.014 to 0.092)
Race = 5, Hispanic	-0.242*** (-0.339 to -0.145)	-0.241*** (-0.339 to -0.144)	-0.240*** (-0.339 to -0.142)	-0.114*** (-0.151 to -0.076)	-0.113*** (-0.151 to -0.076)	-0.113*** (-0.151 to -0.075)	-0.127*** (-0.190 to -0.064)	-0.127*** (-0.190 to -0.064)	-0.126*** (-0.190 to -0.062)	0.002 (-0.021 to 0.025)	0.002 (-0.021 to 0.026)	0.002 (-0.021 to 0.025)
Race = 6, Other/ Mixed/ Missing	0.031 (-0.069 to 0.130)	0.031 (-0.068 to 0.131)	0.032 (-0.068 to 0.133)	-0.011 (-0.051 to 0.029)	-0.011 (-0.052 to 0.030)	-0.010 (-0.051 to 0.032)	0.001 (-0.065 to 0.067)	0.002 (-0.064 to 0.068)	0.002 (-0.064 to 0.069)	0.043*** (0.019 to 0.068)	0.043*** (0.019 to 0.068)	0.042*** (0.018 to 0.067)
Marital status = 1, Married	-0.670***	-0.670***	-0.670***	-0.296***	-0.297***	-0.297***	-0.283***	-0.283***	-0.283***	-0.091***	-0.091***	-0.091***

© 2023 Rokicki S et al. *JAMA Network Open.*

VARIABLES	(1)	(2)	(3)	(4)	(5)	(6)	(7)	(8)	(9)	(10)	(11)	(12)
	Total number of stressors			Total partner stress			Total financial stress			Total traumatic stress		
	Indiv controls	State controls	State time trends	Indiv controls	State controls	State time trends	Indiv controls	State controls	State time trends	Indiv controls	State controls	State time trends
	(-0.753 to -0.588)	(-0.753 to -0.588)	(-0.753 to -0.587)	(-0.330 to -0.263)	(-0.330 to -0.263)	(-0.330 to -0.263)	(-0.330 to -0.237)	(-0.330 to -0.237)	(-0.330 to -0.236)	(-0.105 to -0.078)	(-0.105 to -0.078)	(-0.105 to -0.078)
Nulliparous = 1, Yes	-0.171***	-0.171***	-0.171***	-0.073***	-0.073***	-0.073***	-0.077***	-0.077***	-0.077***	-0.021***	-0.021***	-0.021***
	(-0.203 to -0.139)	(-0.203 to -0.139)	(-0.203 to -0.139)	(-0.087 to -0.059)	(-0.087 to -0.059)	(-0.086 to -0.059)	(-0.098 to -0.056)	(-0.098 to -0.056)	(-0.098 to -0.056)	(-0.028 to -0.014)	(-0.028 to -0.014)	(-0.028 to -0.014)
Nulliparous = 99, Missing	-0.262***	-0.265***	-0.269***	-0.126***	-0.126***	-0.128***	-0.146***	-0.147***	-0.149***	0.012	0.012	0.012
	(-0.337 to -0.188)	(-0.336 to -0.194)	(-0.340 to -0.198)	(-0.159 to -0.093)	(-0.159 to -0.094)	(-0.161 to -0.095)	(-0.205 to -0.087)	(-0.205 to -0.090)	(-0.207 to -0.092)	(-0.011 to 0.036)	(-0.011 to 0.035)	(-0.011 to 0.035)
Education = 2, HS Diploma	0.039	0.038	0.038	0.022**	0.022**	0.022**	0.055***	0.055***	0.055***	-0.040***	-0.040***	-0.040***
	(-0.009 to 0.087)	(-0.009 to 0.086)	(-0.010 to 0.086)	(0.002 to 0.043)	(0.002 to 0.043)	(0.002 to 0.043)	(0.024 to 0.086)	(0.024 to 0.085)	(0.024 to 0.085)	(-0.048 to -0.032)	(-0.048 to -0.032)	(-0.048 to -0.032)
Month of Birth = 2, 2	0.002	0.002	0.001	-0.002	-0.002	-0.003	0.001	0.001	0.001	0.003	0.003	0.003
	(-0.056 to 0.060)	(-0.056 to 0.060)	(-0.057 to 0.058)	(-0.024 to 0.020)	(-0.024 to 0.019)	(-0.024 to 0.019)	(-0.037 to 0.039)	(-0.037 to 0.039)	(-0.037 to 0.039)	(-0.011 to 0.018)	(-0.011 to 0.018)	(-0.012 to 0.017)
Month of Birth = 3, 3	0.004	0.005	0.006	-0.011	-0.010	-0.010	0.015	0.016	0.017	-0.001	-0.001	-0.001
	(-0.040 to 0.049)	(-0.040 to 0.050)	(-0.038 to 0.051)	(-0.040 to 0.019)	(-0.040 to 0.019)	(-0.039 to 0.019)	(-0.010 to 0.040)	(-0.009 to 0.041)	(-0.008 to 0.042)	(-0.014 to 0.012)	(-0.014 to 0.012)	(-0.014 to 0.012)
Month of Birth = 4, 4	-0.015	-0.014	-0.015	0.002	0.002	0.002	-0.012	-0.012	-0.011	-0.005	-0.005	-0.005
	(-0.065 to 0.034)	(-0.064 to 0.036)	(-0.064 to 0.035)	(-0.024 to 0.028)	(-0.024 to 0.029)	(-0.024 to 0.028)	(-0.050 to 0.025)	(-0.049 to 0.026)	(-0.048 to 0.025)	(-0.016 to 0.006)	(-0.016 to 0.005)	(-0.016 to 0.005)
Month of Birth = 5, 5	-0.037	-0.036	-0.037	-0.007	-0.006	-0.007	-0.032	-0.031	-0.032	0.001	0.001	0.001
	(-0.098 to 0.024)	(-0.097 to 0.026)	(-0.098 to 0.024)	(-0.030 to 0.016)	(-0.029 to 0.017)	(-0.030 to 0.016)	(-0.071 to 0.007)	(-0.070 to 0.007)	(-0.070 to 0.007)	(-0.011 to 0.013)	(-0.011 to 0.013)	(-0.011 to 0.013)
Month of Birth = 6, 6	-0.020	-0.019	-0.017	-0.002	-0.002	-0.001	-0.010	-0.009	-0.009	-0.008	-0.008	-0.007
	(-0.063 to 0.023)	(-0.062 to 0.024)	(-0.061 to 0.026)	(-0.018 to 0.013)	(-0.017 to 0.013)	(-0.016 to 0.013)	(-0.051 to 0.030)	(-0.049 to 0.031)	(-0.049 to 0.032)	(-0.021 to 0.006)	(-0.021 to 0.006)	(-0.021 to 0.006)
Month of Birth = 7, 7	-0.068	-0.065	-0.065	-0.023*	-0.022	-0.022	-0.040	-0.038	-0.038	-0.005	-0.005	-0.005
	(-0.151 to 0.016)	(-0.148 to 0.019)	(-0.149 to 0.020)	(-0.049 to 0.004)	(-0.048 to 0.005)	(-0.049 to 0.005)	(-0.099 to 0.020)	(-0.097 to 0.021)	(-0.097 to 0.021)	(-0.018 to 0.007)	(-0.018 to 0.007)	(-0.018 to 0.007)

© 2023 Rokicki S et al. *JAMA Network Open.*

VARIABLES	(1) Total number of stressors: Indiv controls	(2) Total number of stressors: State controls	(3) Total number of stressors: State time trends	(4) Total partner stress: Indiv controls	(5) Total partner stress: State controls	(6) Total partner stress: State time trends	(7) Total financial stress: Indiv controls	(8) Total financial stress: State controls	(9) Total financial stress: State time trends	(10) Total traumatic stress: Indiv controls	(11) Total traumatic stress: State controls	(12) Total traumatic stress: State time trends
Month of Birth = 8, 8	-0.046 (-0.117 to 0.026)	-0.044 (-0.115 to 0.026)	-0.043 (-0.115 to 0.028)	-0.013 (-0.037 to 0.012)	-0.012 (-0.036 to 0.012)	-0.012 (-0.036 to 0.013)	-0.031 (-0.083 to 0.021)	-0.030 (-0.081 to 0.022)	-0.029 (-0.081 to 0.024)	-0.003 (-0.013 to 0.008)	-0.003 (-0.013 to 0.008)	-0.003 (-0.013 to 0.008)
Month of Birth = 9, 9	-0.042 (-0.101 to 0.017)	-0.040 (-0.098 to 0.019)	-0.036 (-0.094 to 0.022)	-0.022* (-0.048 to 0.004)	-0.021* (-0.046 to 0.004)	-0.020 (-0.045 to 0.005)	-0.013 (-0.053 to 0.027)	-0.012 (-0.051 to 0.028)	-0.009 (-0.049 to 0.030)	-0.007 (-0.020 to 0.007)	-0.007 (-0.020 to 0.007)	-0.007 (-0.020 to 0.007)
Month of Birth = 10, 10	-0.046 (-0.118 to 0.026)	-0.044 (-0.117 to 0.029)	-0.040 (-0.114 to 0.034)	-0.020 (-0.051 to 0.010)	-0.019 (-0.050 to 0.012)	-0.018 (-0.049 to 0.014)	-0.015 (-0.058 to 0.029)	-0.013 (-0.057 to 0.030)	-0.011 (-0.055 to 0.034)	-0.012* (-0.026 to 0.002)	-0.012 (-0.027 to 0.003)	-0.012* (-0.026 to 0.002)
Month of Birth = 11, 11	-0.039 (-0.102 to 0.025)	-0.036 (-0.100 to 0.029)	-0.032 (-0.096 to 0.032)	-0.009 (-0.034 to 0.017)	-0.008 (-0.034 to 0.018)	-0.007 (-0.033 to 0.018)	-0.024 (-0.076 to 0.029)	-0.022 (-0.075 to 0.031)	-0.020 (-0.073 to 0.033)	-0.007 (-0.018 to 0.004)	-0.006 (-0.018 to 0.005)	-0.006 (-0.018 to 0.005)
Month of Birth = 12, 12	-0.063 (-0.140 to 0.014)	-0.060 (-0.137 to 0.017)	-0.056 (-0.132 to 0.020)	-0.026** (-0.052 to -0.001)	-0.025** (-0.050 to -0.000)	-0.024* (-0.050 to 0.001)	-0.028 (-0.084 to 0.029)	-0.026 (-0.082 to 0.030)	-0.023 (-0.079 to 0.032)	-0.010* (-0.020 to 0.001)	-0.010* (-0.021 to 0.001)	-0.009* (-0.020 to 0.001)
year = 2005	-0.043 (-0.100 to 0.014)	-0.051* (-0.111 to 0.009)	-0.037 (-0.091 to 0.018)	-0.009 (-0.025 to 0.007)	-0.010 (-0.027 to 0.007)	-0.010 (-0.030 to 0.009)	-0.028 (-0.064 to 0.007)	-0.036* (-0.073 to 0.000)	-0.024 (-0.054 to 0.005)	-0.005 (-0.020 to 0.010)	-0.004 (-0.020 to 0.011)	-0.001 (-0.018 to 0.015)
year = 2006	-0.072*** (-0.107 to -0.038)	-0.106*** (-0.156 to -0.057)	-0.116*** (-0.162 to -0.069)	-0.029** (-0.057 to -0.001)	-0.039*** (-0.068 to -0.011)	-0.046** (-0.080 to -0.012)	-0.037*** (-0.064 to -0.010)	-0.059*** (-0.095 to -0.024)	-0.057*** (-0.094 to -0.020)	-0.006 (-0.021 to 0.010)	-0.008 (-0.026 to 0.010)	-0.013 (-0.035 to 0.009)
year = 2007	-0.100*** (-0.152 to -0.048)	-0.160*** (-0.223 to -0.096)	-0.179*** (-0.241 to -0.117)	-0.050*** (-0.070 to -0.030)	-0.071*** (-0.097 to -0.046)	-0.077*** (-0.104 to -0.050)	-0.051*** (-0.081 to -0.021)	-0.085*** (-0.122 to -0.048)	-0.088*** (-0.130 to -0.046)	0.000 (-0.017 to 0.018)	-0.004 (-0.023 to 0.014)	-0.015 (-0.038 to 0.007)
year = 2008	-0.066** (-0.126 to -0.007)	-0.154*** (-0.261 to -0.047)	-0.179*** (-0.274 to -0.084)	-0.057*** (-0.089 to -0.025)	-0.087*** (-0.137 to -0.037)	-0.097*** (-0.132 to -0.061)	-0.029 (-0.069 to 0.011)	-0.080*** (-0.139 to -0.021)	-0.083*** (-0.137 to -0.029)	0.020** (0.001 to 0.038)	0.012 (-0.013 to 0.036)	-0.001 (-0.031 to 0.030)
year = 2009	-0.035 (-0.107 to 0.036)	-0.113*** (-0.184 to -0.041)	-0.137*** (-0.221 to -0.053)	-0.064*** (-0.085 to -0.042)	-0.092*** (-0.127 to -0.057)	-0.105*** (-0.138 to -0.072)	0.020 (-0.037 to 0.077)	-0.024 (-0.073 to 0.025)	-0.019 (-0.073 to 0.034)	0.008 (-0.007 to 0.023)	0.002 (-0.019 to 0.022)	-0.015 (-0.045 to 0.016)
year = 2010	-0.097*** (-0.156 to -0.039)	-0.143*** (-0.216 to -0.070)	-0.167*** (-0.248 to -0.086)	-0.064*** (-0.087 to -0.041)	-0.080*** (-0.108 to -0.052)	-0.108*** (-0.139 to -0.076)	-0.026 (-0.066 to 0.015)	-0.058** (-0.111 to -0.006)	-0.041 (-0.100 to 0.019)	-0.009 (-0.033 to 0.016)	-0.007 (-0.036 to 0.021)	-0.020 (-0.047 to 0.007)

© 2023 Rokicki S et al. *JAMA Network Open.*

VARIABLES	(1) Total number of stressors, Indiv controls	(2) Total number of stressors, State controls	(3) Total number of stressors, State time trends	(4) Total partner stress, Indiv controls	(5) Total partner stress, State controls	(6) Total partner stress, State time trends	(7) Total financial stress, Indiv controls	(8) Total financial stress, State controls	(9) Total financial stress, State time trends	(10) Total traumatic stress, Indiv controls	(11) Total traumatic stress, State controls	(12) Total traumatic stress, State time trends
year = 2011	-0.213*** (-0.290 to -0.137)	-0.133** (-0.249 to -0.017)	-0.157*** (-0.236 to -0.077)	-0.102*** (-0.136 to -0.069)	-0.065*** (-0.106 to -0.024)	-0.134*** (-0.171 to -0.097)	-0.106*** (-0.154 to -0.059)	-0.092** (-0.182 to -0.002)	-0.043 (-0.101 to 0.014)	-0.005 (-0.028 to 0.017)	0.023 (-0.012 to 0.058)	0.021* (-0.001 to 0.042)
year = 2012	-0.226*** (-0.336 to -0.116)	-0.140* (-0.280 to 0.001)	-0.195*** (-0.300 to -0.089)	-0.096*** (-0.140 to -0.051)	-0.058** (-0.105 to -0.011)	-0.139*** (-0.180 to -0.098)	-0.101*** (-0.161 to -0.041)	-0.086 (-0.196 to 0.024)	-0.051 (-0.128 to 0.027)	-0.030** (-0.060 to -0.000)	0.002 (-0.039 to 0.044)	-0.005 (-0.032 to 0.022)
year = 2013	-0.289*** (-0.386 to -0.192)	-0.261*** (-0.396 to -0.126)	-0.327*** (-0.410 to -0.244)	-0.117*** (-0.147 to -0.088)	-0.099*** (-0.133 to -0.064)	-0.183*** (-0.218 to -0.147)	-0.133*** (-0.198 to -0.068)	-0.149*** (-0.253 to -0.046)	-0.126*** (-0.191 to -0.061)	-0.040*** (-0.068 to -0.011)	-0.015 (-0.055 to 0.024)	-0.019 (-0.046 to 0.008)
year = 2014	-0.362*** (-0.429 to -0.295)	-0.375*** (-0.524 to -0.225)	-0.491*** (-0.599 to -0.384)	-0.154*** (-0.184 to -0.123)	-0.151*** (-0.195 to -0.108)	-0.253*** (-0.283 to -0.223)	-0.165*** (-0.205 to -0.125)	-0.202*** (-0.312 to -0.091)	-0.204*** (-0.284 to -0.124)	-0.045*** (-0.070 to -0.019)	-0.025 (-0.056 to 0.006)	-0.036** (-0.064 to -0.007)
year = 2015	-0.326*** (-0.390 to -0.262)	-0.368*** (-0.509 to -0.227)	-0.494*** (-0.585 to -0.403)	-0.137*** (-0.165 to -0.109)	-0.147*** (-0.187 to -0.108)	-0.252*** (-0.287 to -0.217)	-0.147*** (-0.190 to -0.105)	-0.197*** (-0.312 to -0.082)	-0.211*** (-0.295 to -0.126)	-0.042*** (-0.067 to -0.017)	-0.026 (-0.059 to 0.007)	-0.032** (-0.061 to -0.003)
year = o,			0.000 (0.000 to 0.000)			0.000 (0.000 to 0.000)			0.000 (0.000 to 0.000)			0.000 (0.000 to 0.000)
STATE NAME = 2, AL	-0.006 (-0.081 to 0.068)	0.192 (-0.397 to 0.782)	441.463*** (336.134 to 546.793)	-0.003 (-0.029 to 0.022)	0.072 (-0.139 to 0.282)	139.884*** (86.117 to 193.651)	0.028 (-0.018 to 0.075)	0.118 (-0.316 to 0.553)	261.086*** (191.282 to 330.891)	-0.032** (-0.060 to -0.004)	0.000 (-0.106 to 0.106)	44.306*** (12.886 to 75.727)
STATE NAME = 3, AR	0.341*** (0.277 to 0.406)	0.490* (-0.057 to 1.036)	2.567 (-25.349 to 30.482)	0.130*** (0.110 to 0.150)	0.195* (-0.002 to 0.391)	-16.047*** (-24.793 to -7.302)	0.225*** (0.181 to 0.268)	0.281 (-0.146 to 0.707)	2.133 (-17.077 to 21.343)	-0.014 (-0.035 to 0.008)	0.015 (-0.093 to 0.123)	16.771*** (7.881 to 25.661)
STATE NAME = 5, CO	0.261*** (0.188 to 0.334)	0.190 (-0.271 to 0.650)	-30.652 (-83.320 to 22.017)	0.090*** (0.069 to 0.111)	0.100 (-0.066 to 0.265)	-56.993*** (-84.137 to -29.848)	0.177*** (0.126 to 0.227)	0.099 (-0.246 to 0.445)	1.175 (-33.739 to 36.090)	-0.006 (-0.025 to 0.014)	-0.012 (-0.126 to 0.102)	27.731*** (11.577 to 43.884)
STATE NAME = 6, CT	0.126*** (0.055 to 0.198)	-0.132 (-0.519 to 0.254)	-172.055*** (-284.721 to -59.389)	0.037** (0.008 to 0.066)	0.016 (-0.106 to 0.138)	24.022 (-26.362 to 74.405)	0.128*** (0.083 to 0.174)	-0.070 (-0.370 to 0.229)	-143.236*** (-224.781 to -61.691)	-0.038*** (-0.058 to -0.019)	-0.078 (-0.188 to 0.032)	-48.207*** (-81.146 to -15.268)
STATE NAME = 8, DE	0.162*** (0.104 to 0.220)	0.069 (-0.387 to 0.524)	13.272 (-20.665 to 47.209)	0.049*** (0.026 to 0.072)	0.060 (-0.083 to 0.204)	-9.202 (-20.672 to 2.268)	0.111*** (0.069 to 0.152)	0.031 (-0.345 to 0.407)	11.654 (-12.589 to 35.896)	0.003 (-0.015 to 0.020)	-0.022 (-0.136 to 0.091)	12.510** (2.128 to 22.893)

© 2023 Rokicki S et al. *JAMA Network Open.*

VARIABLES	(1)	(2)	(3)	(4)	(5)	(6)	(7)	(8)	(9)	(10)	(11)	(12)
	Total number of stressors			Total partner stress			Total financial stress			Total traumatic stress		
	Indiv controls	State controls	State time trends	Indiv controls	State controls	State time trends	Indiv controls	State controls	State time trends	Indiv controls	State controls	State time trends
STATE NAME = 9, FL	0.134***	-0.136	397.819***	0.047***	0.015	64.626***	0.105***	-0.138	245.601***	-0.019*	-0.020	91.923***
	(0.045 to 0.224)	(-0.695 to 0.424)	(273.896 to 521.741)	(0.026 to 0.069)	(-0.214 to 0.244)	(19.690 to 109.561)	(0.046 to 0.165)	(-0.534 to 0.258)	(142.407 to 348.795)	(-0.039 to 0.002)	(-0.183 to 0.142)	(57.891 to 125.954)
STATE NAME = 10, GA	-0.193***	-0.301	128.818***	-0.101***	-0.093	50.925***	-0.054**	-0.182	54.157***	-0.039***	-0.030	24.730**
	(-0.264 to -0.122)	(-0.810 to 0.207)	(73.394 to 184.241)	(-0.122 to -0.079)	(-0.302 to 0.115)	(31.764 to 70.087)	(-0.105 to -0.003)	(-0.558 to 0.193)	(16.794 to 91.520)	(-0.062 to -0.016)	(-0.160 to 0.101)	(5.938 to 43.522)
STATE NAME = 11, HI	0.007	-0.050	54.168***	0.055***	0.027	7.895	-0.023	-0.038	28.537**	-0.026***	-0.041	18.049***
	(-0.062 to 0.076)	(-0.230 to 0.130)	(19.809 to 88.527)	(0.031 to 0.080)	(-0.032 to 0.086)	(-3.432 to 19.222)	(-0.065 to 0.018)	(-0.187 to 0.111)	(5.516 to 51.558)	(-0.043 to -0.009)	(-0.096 to 0.015)	(8.782 to 27.316)
STATE NAME = 12, IA	0.107***	-0.112	-105.204***	0.035***	0.000	-125.560***	0.072***	-0.063	-7.168	-0.000	-0.047	29.294**
	(0.036 to 0.178)	(-0.551 to 0.326)	(-163.677 to -46.730)	(0.010 to 0.059)	(-0.133 to 0.133)	(-152.782 to -98.338)	(0.028 to 0.117)	(-0.412 to 0.285)	(-46.005 to 31.669)	(-0.026 to 0.026)	(-0.160 to 0.066)	(3.446 to 55.143)
STATE NAME = 13, IL	0.138***	-0.108	63.773*	0.070***	0.044	3.183	0.064***	-0.162	6.198	0.004	0.003	57.591***
	(0.085 to 0.190)	(-0.600 to 0.384)	(-3.009 to 130.555)	(0.053 to 0.087)	(-0.155 to 0.242)	(-22.877 to 29.243)	(0.028 to 0.100)	(-0.528 to 0.204)	(-41.639 to 54.035)	(-0.010 to 0.017)	(-0.144 to 0.151)	(34.607 to 80.575)
STATE NAME = 17, LA	0.114***	0.210	27.909*	0.080***	0.137	5.559	0.076***	0.085	3.557	-0.042***	-0.012	19.666***
	(0.047 to 0.181)	(-0.359 to 0.779)	(-4.067 to 59.885)	(0.060 to 0.099)	(-0.074 to 0.348)	(-3.357 to 14.475)	(0.032 to 0.120)	(-0.349 to 0.519)	(-18.890 to 26.004)	(-0.064 to -0.020)	(-0.131 to 0.107)	(8.682 to 30.651)
STATE NAME = 18, MA	0.144***	-0.116	65.269***	0.065***	0.019	-7.899	0.086***	-0.112	44.207***	-0.005	-0.026	31.977***
	(0.094 to 0.193)	(-0.417 to 0.185)	(24.707 to 105.831)	(0.045 to 0.084)	(-0.094 to 0.132)	(-30.098 to 14.301)	(0.054 to 0.117)	(-0.348 to 0.123)	(11.622 to 76.792)	(-0.019 to 0.008)	(-0.129 to 0.077)	(18.524 to 45.431)
STATE NAME = 19, MD	0.109***	-0.247	16.897	0.012	-0.039	-7.873	0.117***	-0.144	-12.712	-0.020*	-0.067	38.969***
	(0.043 to 0.175)	(-0.673 to 0.178)	(-30.917 to 64.712)	(-0.008 to 0.032)	(-0.185 to 0.107)	(-23.139 to 7.394)	(0.069 to 0.165)	(-0.467 to 0.180)	(-47.974 to 22.550)	(-0.041 to 0.002)	(-0.191 to 0.057)	(24.223 to 53.715)
STATE NAME = 20, ME	0.232***	0.227	-23.786*	0.055***	0.067	-13.789***	0.171***	0.163	-13.767*	0.006	-0.002	3.835
	(0.186 to 0.279)	(-0.153 to 0.607)	(-48.523 to 0.952)	(0.037 to 0.074)	(-0.055 to 0.188)	(-21.453 to -6.125)	(0.144 to 0.198)	(-0.157 to 0.484)	(-30.156 to 2.623)	(-0.009 to 0.022)	(-0.093 to 0.089)	(-3.310 to 10.979)
STATE NAME = 21, MI	0.129***	0.098	-34.886	0.041***	0.068	-13.226**	0.098***	0.015	-28.664*	-0.010	0.011	8.157

© 2023 Rokicki S et al. *JAMA Network Open.*

VARIABLES	(1)	(2)	(3)	(4)	(5)	(6)	(7)	(8)	(9)	(10)	(11)	(12)
	Total number of stressors			Total partner stress			Total financial stress			Total traumatic stress		
	Indiv controls	State controls	State time trends	Indiv controls	State controls	State time trends	Indiv controls	State controls	State time trends	Indiv controls	State controls	State time trends
	(0.078 to 0.179)	(-0.283 to 0.479)	(-79.788 to 10.017)	(0.023 to 0.060)	(-0.095 to 0.232)	(-26.129 to -0.323)	(0.066 to 0.130)	(-0.267 to 0.297)	(-58.536 to 1.209)	(-0.027 to 0.007)	(-0.087 to 0.110)	(-4.627 to 20.941)
STATE NAME = 22, MN	0.014	-0.541**	8.764	-0.012	-0.103	-10.737*	0.035*	-0.350*	5.814	-0.009	-0.090	14.421***
	(-0.045 to 0.073)	(-1.056 to -0.025)	(-22.731 to 40.260)	(-0.030 to 0.006)	(-0.286 to 0.079)	(-22.964 to 1.489)	(-0.005 to 0.075)	(-0.718 to 0.018)	(-16.529 to 28.157)	(-0.028 to 0.010)	(-0.237 to 0.058)	(3.933 to 24.909)
STATE NAME = 23, MO	0.314***	0.356	-40.619**	0.098***	0.138	-35.066***	0.208***	0.194	-16.606	0.007	0.022	11.796**
	(0.254 to 0.373)	(-0.149 to 0.862)	(-80.229 to -1.010)	(0.076 to 0.120)	(-0.055 to 0.331)	(-49.407 to -20.724)	(0.169 to 0.248)	(-0.186 to 0.574)	(-45.995 to 12.783)	(-0.015 to 0.029)	(-0.086 to 0.131)	(0.984 to 22.609)
STATE NAME = 24, MS	0.005	0.195	44.659**	0.076***	0.163	2.911	0.000	0.055	32.524**	-0.070***	-0.021	9.811*
	(-0.075 to 0.085)	(-0.417 to 0.806)	(4.906 to 84.413)	(0.052 to 0.100)	(-0.071 to 0.396)	(-13.240 to 19.062)	(-0.056 to 0.056)	(-0.396 to 0.506)	(6.105 to 58.943)	(-0.094 to -0.047)	(-0.132 to 0.089)	(-0.721 to 20.343)
STATE NAME = 26, NC	0.040	-0.001	90.017***	0.005	0.023	13.828	0.066**	-0.016	59.483***	-0.029***	-0.010	18.514*
	(-0.033 to 0.113)	(-0.508 to 0.506)	(48.084 to 131.950)	(-0.016 to 0.026)	(-0.178 to 0.223)	(-9.973 to 37.629)	(0.013 to 0.118)	(-0.395 to 0.364)	(27.407 to 91.558)	(-0.050 to -0.008)	(-0.133 to 0.114)	(-2.210 to 39.238)
STATE NAME = 28, NE	0.181***	0.068	18.450	0.044***	0.031	-16.260***	0.148***	0.076	18.437**	-0.011	-0.040	17.279***
	(0.108 to 0.253)	(-0.373 to 0.509)	(-5.088 to 41.988)	(0.023 to 0.064)	(-0.111 to 0.173)	(-25.330 to -7.189)	(0.096 to 0.199)	(-0.278 to 0.431)	(0.767 to 36.106)	(-0.033 to 0.012)	(-0.155 to 0.076)	(7.971 to 26.587)
STATE NAME = 29, NH	0.414***	0.378***	-147.964***	0.065***	0.042	35.311**	0.361***	0.374***	-112.255***	-0.011	-0.035	-64.094***
	(0.345 to 0.483)	(0.135 to 0.621)	(-216.557 to -79.370)	(0.041 to 0.090)	(-0.023 to 0.107)	(4.395 to 66.227)	(0.318 to 0.404)	(0.178 to 0.570)	(-155.349 to -69.161)	(-0.038 to 0.017)	(-0.085 to 0.015)	(-94.735 to -33.452)
STATE NAME = 30, NJ	0.035	-0.329	1.649	0.026***	-0.016	-13.194	0.045**	-0.244	-15.376	-0.035***	-0.072	32.230***
	(-0.026 to 0.096)	(-0.815 to 0.157)	(-52.285 to 55.583)	(0.008 to 0.045)	(-0.194 to 0.161)	(-32.553 to 6.165)	(0.001 to 0.090)	(-0.608 to 0.120)	(-51.508 to 20.756)	(-0.051 to -0.019)	(-0.220 to 0.076)	(14.712 to 49.749)
STATE NAME = 31, NM	0.064**	0.094	-26.003	0.030***	0.061	-24.290***	0.053***	0.034	-9.460	-0.018**	0.001	9.205*
	(0.012 to 0.117)	(-0.356 to 0.544)	(-61.411 to 9.405)	(0.011 to 0.049)	(-0.093 to 0.214)	(-39.219 to -9.360)	(0.015 to 0.090)	(-0.323 to 0.390)	(-31.531 to 12.611)	(-0.033 to -0.002)	(-0.096 to 0.097)	(-1.146 to 19.555)
STATE NAME = 32, NY	-0.021	-0.757***	181.474***	-0.009	-0.150	13.444	0.025	-0.559***	72.851*	-0.035***	-0.061	99.319***
	(-0.075 to 0.033)	(-1.289 to -0.225)	(71.589 to 291.359)	(-0.027 to 0.008)	(-0.369 to 0.069)	(-43.604 to 70.493)	(-0.012 to 0.062)	(-0.964 to -0.154)	(-13.016 to 158.719)	(-0.052 to -0.018)	(-0.263 to 0.141)	(56.230 to 142.408)

© 2023 Rokicki S et al. *JAMA Network Open.*

	(1)	(2)	(3)	(4)	(5)	(6)	(7)	(8)	(9)	(10)	(11)	(12)
VARIABLES	Total number of stressors			Total partner stress			Total financial stress			Total traumatic stress		
	Indiv controls	State controls	State time trends	Indiv controls	State controls	State time trends	Indiv controls	State controls	State time trends	Indiv controls	State controls	State time trends
STATE NAME = 33, OH	0.204*** (0.145 to 0.264)	0.090 (-0.346 to 0.526)	21.101 (-28.630 to 70.831)	0.071*** (0.053 to 0.090)	0.068 (-0.115 to 0.252)	-19.618* (-42.696 to 3.460)	0.150*** (0.113 to 0.187)	0.021 (-0.299 to 0.341)	16.303 (-18.963 to 51.570)	-0.016 (-0.038 to 0.005)	-0.005 (-0.123 to 0.114)	27.231*** (9.471 to 44.992)
STATE NAME = 34, OK	0.285*** (0.221 to 0.350)	0.269 (-0.224 to 0.761)	36.871** (8.364 to 65.378)	0.087*** (0.067 to 0.107)	0.104 (-0.067 to 0.276)	-1.271 (-10.711 to 8.170)	0.210*** (0.165 to 0.256)	0.174 (-0.218 to 0.565)	21.001** (0.778 to 41.224)	-0.011 (-0.032 to 0.011)	-0.009 (-0.123 to 0.104)	19.108*** (8.053 to 30.163)
STATE NAME = 35, OR	0.221*** (0.174 to 0.267)	0.269 (-0.110 to 0.649)	58.352*** (31.092 to 85.612)	0.033*** (0.014 to 0.052)	0.078 (-0.056 to 0.211)	-18.915*** (-27.072 to -10.757)	0.160*** (0.133 to 0.187)	0.149 (-0.137 to 0.436)	46.725*** (29.201 to 64.249)	0.027*** (0.011 to 0.042)	0.041 (-0.043 to 0.125)	31.602*** (23.507 to 39.698)
STATE NAME = 36, PA	0.031 (-0.029 to 0.090)	-0.151 (-0.614 to 0.311)	-18.481 (-83.306 to 46.345)	0.046*** (0.023 to 0.068)	0.024 (-0.171 to 0.218)	-37.898*** (-65.350 to -10.445)	0.014 (-0.027 to 0.055)	-0.154 (-0.493 to 0.184)	-1.448 (-49.587 to 46.690)	-0.029*** (-0.050 to -0.008)	-0.026 (-0.160 to 0.107)	23.148* (-0.794 to 47.089)
STATE NAME = 37, RI	0.099*** (0.053 to 0.145)	-0.010 (-0.331 to 0.310)	-56.151*** (-93.861 to -18.441)	0.057*** (0.040 to 0.073)	0.064 (-0.043 to 0.172)	-19.514*** (-31.990 to -7.037)	0.080*** (0.052 to 0.108)	-0.015 (-0.281 to 0.251)	-37.753*** (-64.121 to -11.385)	-0.038*** (-0.051 to -0.025)	-0.059 (-0.148 to 0.031)	1.137 (-11.604 to 13.879)
STATE NAME = 38, SC	0.147*** (0.073 to 0.221)	0.229 (-0.308 to 0.766)	-186.263*** (-239.244 to -133.282)	0.048*** (0.028 to 0.069)	0.103 (-0.096 to 0.302)	-48.374*** (-65.793 to -30.954)	0.131*** (0.082 to 0.180)	0.138 (-0.276 to 0.551)	-71.179*** (-111.707 to -30.651)	-0.031*** (-0.052 to -0.010)	-0.010 (-0.124 to 0.104)	-66.488*** (-83.493 to -49.483)
STATE NAME = 40, TN	0.237*** (0.163 to 0.312)	0.278 (-0.276 to 0.833)	8.980 (-51.923 to 69.883)	0.056*** (0.030 to 0.082)	0.097 (-0.112 to 0.307)	-37.665** (-69.488 to -5.841)	0.183*** (0.131 to 0.235)	0.164 (-0.270 to 0.597)	25.474 (-18.805 to 69.752)	0.000 (-0.025 to 0.026)	0.019 (-0.103 to 0.142)	26.118** (5.684 to 46.553)
STATE NAME = 41, TX	0.157*** (0.055 to 0.260)	-0.437 (-1.190 to 0.315)	183.140** (39.087 to 327.193)	0.072*** (0.046 to 0.097)	-0.032 (-0.361 to 0.298)	9.031 (-62.825 to 80.887)	0.095** (0.018 to 0.173)	-0.416 (-0.928 to 0.096)	39.938 (-69.786 to 149.661)	-0.011 (-0.040 to 0.018)	-0.006 (-0.258 to 0.245)	141.690*** (79.135 to 204.244)
STATE NAME = 42, UT	0.162*** (0.083 to 0.240)	0.141 (-0.249 to 0.530)	5.186 (-17.936 to 28.309)	0.041*** (0.018 to 0.064)	0.048 (-0.089 to 0.184)	-23.765*** (-31.289 to -16.241)	0.140*** (0.088 to 0.192)	0.122 (-0.171 to 0.414)	12.501 (-4.441 to 29.443)	-0.019 (-0.042 to 0.004)	-0.030 (-0.122 to 0.061)	17.458*** (8.771 to 26.146)
STATE NAME = 44, VT	0.208***	-0.122	-41.280***	0.062***	0.006	-21.180***	0.136***	-0.076	-15.787**	0.010	-0.052	-4.361

© 2023 Rokicki S et al. *JAMA Network Open.*

VARIABLES	(1) Total number of stressors Indiv controls	(2) State controls	(3) State time trends	(4) Total partner stress Indiv controls	(5) State controls	(6) State time trends	(7) Total financial stress Indiv controls	(8) State controls	(9) State time trends	(10) Total traumatic stress Indiv controls	(11) State controls	(12) State time trends
	(0.159 to 0.256)	(-0.477 to 0.234)	(-58.217 to -24.343)	(0.042 to 0.082)	(-0.130 to 0.143)	(-25.705 to -16.655)	(0.109 to 0.163)	(-0.339 to 0.186)	(-28.764 to -2.809)	(-0.005 to 0.024)	(-0.159 to 0.055)	(-9.801 to 1.079)
STATE NAME = 45, WA	0.203***	0.185	62.136***	0.061***	0.077	-10.681	0.118***	0.070	34.810**	0.023***	0.034	39.947***
	(0.154 to 0.252)	(-0.180 to 0.550)	(16.311 to 107.961)	(0.041 to 0.081)	(-0.060 to 0.215)	(-31.972 to 10.610)	(0.089 to 0.146)	(-0.202 to 0.341)	(0.360 to 69.260)	(0.007 to 0.039)	(-0.059 to 0.127)	(22.408 to 57.486)
STATE NAME = 46, WI	0.119***	-0.081	13.543	0.048***	0.000	-53.049**	0.076***	-0.056	35.959	-0.004	-0.026	34.483***
	(0.060 to 0.178)	(-0.342 to 0.180)	(-53.873 to 80.959)	(0.026 to 0.069)	(-0.096 to 0.096)	(-93.633 to -12.466)	(0.034 to 0.118)	(-0.251 to 0.139)	(-9.342 to 81.260)	(-0.023 to 0.015)	(-0.103 to 0.050)	(10.604 to 58.361)
STATE NAME = 47, WV	0.066**	0.258	-71.242***	0.031***	0.085	-23.252***	0.077***	0.181	-53.103***	-0.041***	-0.007	5.330
	(0.001 to 0.131)	(-0.188 to 0.703)	(-99.231 to -43.254)	(0.009 to 0.053)	(-0.067 to 0.236)	(-30.891 to -15.612)	(0.037 to 0.117)	(-0.167 to 0.528)	(-73.514 to -32.692)	(-0.063 to -0.018)	(-0.087 to 0.073)	(-3.555 to 14.215)
STATE NAME = 48, WY	0.147***	0.180	-15.770	0.077***	0.078	-15.707	0.100***	0.140	-7.757	-0.030**	-0.038	8.069
	(0.081 to 0.214)	(-0.163 to 0.522)	(-88.047 to 56.508)	(0.053 to 0.100)	(-0.031 to 0.187)	(-45.524 to 14.110)	(0.052 to 0.148)	(-0.137 to 0.417)	(-63.401 to 47.887)	(-0.053 to -0.006)	(-0.112 to 0.037)	(-6.961 to 23.098)
1b.state#co.year			0.000			0.000			0.000			0.000
			(0.000 to 0.000)			(0.000 to 0.000)			(0.000 to 0.000)			(0.000 to 0.000)
2.state#c.year			-0.219***			-0.069***			-0.130***			-0.022***
			(-0.271 to -0.167)			(-0.096 to -0.043)			(-0.164 to -0.095)			(-0.038 to -0.006)
3.state#c.year			-0.001			0.008***			-0.001			-0.008***
			(-0.015 to 0.013)			(0.004 to 0.012)			(-0.011 to 0.009)			(-0.013 to -0.004)
5.state#c.year			0.015			0.028***			-0.001			-0.014***
			(-0.011 to 0.041)			(0.015 to 0.042)			(-0.018 to 0.017)			(-0.022 to -0.006)
6.state#c.year			0.085***			-0.012			0.071***			0.024***
			(0.029 to 0.141)			(-0.037 to 0.013)			(0.031 to 0.112)			(0.007 to 0.040)
8.state#c.year			-0.006			0.005			-0.006			-0.006**
			(-0.023 to 0.010)			(-0.001 to 0.010)			(-0.018 to 0.006)			(-0.011 to -0.001)
9.state#c.year			-0.199***			-0.032***			-0.123***			-0.046***

© 2023 Rokicki S et al. *JAMA Network Open.*

VARIABLES	(1)<br>Total number of stressors<br>Indiv controls	(2)<br><br>State controls	(3)<br><br>State time trends	(4)<br>Total partner stress<br>Indiv controls	(5)<br><br>State controls	(6)<br><br>State time trends	(7)<br>Total financial stress<br>Indiv controls	(8)<br><br>State controls	(9)<br><br>State time trends	(10)<br>Total traumatic stress<br>Indiv controls	(11)<br><br>State controls	(12)<br><br>State time trends
			(-0.261 to - 0.137)			(-0.055 to - 0.010)			(-0.174 to - 0.071)			(-0.063 to - 0.029)
10.state#c.year			-0.065***<br>(-0.092 to - 0.037)			-0.025***<br>(-0.035 to - 0.016)			-0.027***<br>(-0.046 to - 0.009)			-0.012**<br>(-0.022 to - 0.003)
11.state#c.year			-0.027***<br>(-0.044 to - 0.010)			-0.004<br>(-0.010 to 0.002)			-0.014**<br>(-0.026 to - 0.003)			-0.009***<br>(-0.014 to - 0.004)
12.state#c.year			0.052***<br>(0.023 to 0.081)			0.062***<br>(0.049 to 0.076)			0.003<br>(-0.016 to 0.023)			-0.015**<br>(-0.027 to - 0.002)
13.state#c.year			-0.032*<br>(-0.066 to 0.001)			-0.002<br>(-0.015 to 0.012)			-0.003<br>(-0.027 to 0.021)			-0.029***<br>(-0.041 to - 0.017)
17.state#c.year			-0.014*<br>(-0.030 to 0.002)			-0.003<br>(-0.007 to 0.002)			-0.002<br>(-0.013 to 0.009)			-0.010***<br>(-0.015 to - 0.004)
18.state#c.year			-0.033***<br>(-0.053 to - 0.012)			0.004<br>(-0.007 to 0.015)			-0.022***<br>(-0.039 to - 0.006)			-0.016***<br>(-0.023 to - 0.009)
19.state#c.year			-0.009<br>(-0.032 to 0.015)			0.004<br>(-0.004 to 0.012)			0.006<br>(-0.011 to 0.024)			-0.020***<br>(-0.027 to - 0.012)
20.state#c.year			0.012*<br>(-0.000 to 0.024)			0.007***<br>(0.003 to 0.011)			0.007*<br>(-0.001 to 0.015)			-0.002<br>(-0.005 to 0.002)
21.state#c.year			0.017<br>(-0.005 to 0.039)			0.007**<br>(0.000 to 0.013)			0.014*<br>(-0.001 to 0.029)			-0.004<br>(-0.011 to 0.002)
22.state#c.year			-0.005<br>(-0.020 to 0.011)			0.005*<br>(-0.001 to 0.012)			-0.003<br>(-0.014 to 0.008)			-0.007***<br>(-0.013 to - 0.002)
23.state#c.year			0.020**<br>(0.000 to 0.040)			0.017***<br>(0.010 to 0.025)			0.008<br>(-0.006 to 0.023)			-0.006**<br>(-0.011 to - 0.001)
24.state#c.year			-0.022**			-0.001			-0.016**			-0.005*

© 2023 Rokicki S et al. *JAMA Network Open.*

VARIABLES	(1) Total number of stressors Indiv controls	(2) State controls	(3) State time trends	(4) Total partner stress Indiv controls	(5) State controls	(6) State time trends	(7) Total financial stress Indiv controls	(8) State controls	(9) State time trends	(10) Total traumatic stress Indiv controls	(11) State controls	(12) State time trends
			(-0.042 to -0.002)			(-0.009 to 0.007)			(-0.029 to -0.003)			(-0.010 to 0.000)
26.state#c.year			-0.045*** (-0.066 to -0.024)			-0.007 (-0.019 to 0.005)			-0.030*** (-0.046 to -0.014)			-0.009* (-0.020 to 0.001)
28.state#c.year			-0.009 (-0.021 to 0.003)			0.008*** (0.004 to 0.013)			-0.009** (-0.018 to -0.000)			-0.009*** (-0.013 to -0.004)
29.state#c.year			0.074*** (0.040 to 0.108)			-0.017** (-0.033 to -0.002)			0.056*** (0.035 to 0.077)			0.032*** (0.017 to 0.047)
30.state#c.year			-0.001 (-0.028 to 0.026)			0.007 (-0.003 to 0.016)			0.007 (-0.011 to 0.026)			-0.016*** (-0.025 to -0.007)
31.state#c.year			0.013 (-0.005 to 0.031)			0.012*** (0.005 to 0.020)			0.005 (-0.006 to 0.016)			-0.005* (-0.010 to 0.001)
32.state#c.year			-0.092*** (-0.147 to -0.036)			-0.007 (-0.036 to 0.022)			-0.037* (-0.080 to 0.006)			-0.050*** (-0.072 to -0.028)
33.state#c.year			-0.011 (-0.036 to 0.014)			0.010* (-0.002 to 0.021)			-0.008 (-0.026 to 0.009)			-0.014*** (-0.023 to -0.005)
34.state#c.year			-0.018** (-0.032 to -0.004)			0.001 (-0.004 to 0.005)			-0.010** (-0.020 to -0.000)			-0.010*** (-0.015 to -0.004)
35.state#c.year			-0.029*** (-0.043 to -0.015)			0.009*** (0.005 to 0.013)			-0.023*** (-0.032 to -0.015)			-0.016*** (-0.020 to -0.012)
36.state#c.year			0.009 (-0.024 to 0.041)			0.019*** (0.005 to 0.033)			0.000 (-0.024 to 0.025)			-0.012* (-0.024 to 0.000)
37.state#c.year			0.028*** (0.009 to 0.047)			0.010*** (0.004 to 0.016)			0.019*** (0.006 to 0.032)			-0.001 (-0.007 to 0.006)
38.state#c.year			0.093***			0.024***			0.036***			0.033***

© 2023 Rokicki S et al. *JAMA Network Open.*

VARIABLES	(1) Total number of stressors Indiv controls	(2) Total number of stressors State controls	(3) Total number of stressors State time trends	(4) Total partner stress Indiv controls	(5) Total partner stress State controls	(6) Total partner stress State time trends	(7) Total financial stress Indiv controls	(8) Total financial stress State controls	(9) Total financial stress State time trends	(10) Total traumatic stress Indiv controls	(11) Total traumatic stress State controls	(12) Total traumatic stress State time trends
			(0.067 to 0.119)			(0.015 to 0.033)			(0.015 to 0.056)			(0.025 to 0.042)
40.state#c.year			-0.005 (-0.035 to 0.026)			0.019** (0.003 to 0.035)			-0.013 (-0.035 to 0.009)			-0.013** (-0.023 to -0.003)
41.state#c.year			-0.093** (-0.165 to -0.020)			-0.005 (-0.041 to 0.032)			-0.021 (-0.076 to 0.035)			-0.071*** (-0.103 to -0.040)
42.state#c.year			-0.003 (-0.014 to 0.009)			0.012*** (0.008 to 0.016)			-0.006 (-0.015 to 0.002)			-0.009*** (-0.013 to -0.004)
44.state#c.year			0.021*** (0.012 to 0.029)			0.011*** (0.008 to 0.013)			0.008** (0.001 to 0.014)			0.002 (-0.001 to 0.005)
45.state#c.year			-0.031*** (-0.054 to -0.008)			0.005 (-0.005 to 0.016)			-0.017** (-0.035 to -0.000)			-0.020*** (-0.029 to -0.011)
46.state#c.year			-0.007 (-0.041 to 0.027)			0.026** (0.006 to 0.047)			-0.018 (-0.041 to 0.005)			-0.017*** (-0.029 to -0.005)
47.state#c.year			0.036*** (0.022 to 0.050)			0.012*** (0.008 to 0.015)			0.026*** (0.016 to 0.037)			-0.003 (-0.007 to 0.002)
48.state#c.year			0.008 (-0.028 to 0.044)			0.008 (-0.007 to 0.023)			0.004 (-0.024 to 0.031)			-0.004 (-0.011 to 0.003)
Unemployment rate		-0.039** (-0.078 to -0.001)	-0.036** (-0.063 to -0.008)		-0.017* (-0.034 to 0.001)	-0.008* (-0.017 to 0.001)		-0.016 (-0.039 to 0.007)	-0.024** (-0.042 to -0.005)		-0.007 (-0.016 to 0.003)	-0.004 (-0.014 to 0.005)
Gross State Product		0.000*** (0.000 to 0.000)	0.000*** (0.000 to 0.000)		0.000* (-0.000 to 0.000)	0.000 (-0.000 to 0.000)		0.000*** (0.000 to 0.000)	0.000** (0.000 to 0.000)		0.000 (-0.000 to 0.000)	0.000*** (0.000 to 0.000)
Percent Low Income Uninsured Children		0.009	0.007		0.001	-0.000		0.007	0.008*		0.001	-0.001

© 2023 Rokicki S et al. *JAMA Network Open.*

	(1)	(2)	(3)	(4)	(5)	(6)	(7)	(8)	(9)	(10)	(11)	(12)
VARIABLES	Total number of stressors			Total partner stress			Total financial stress			Total traumatic stress		
	Indiv controls	State controls	State time trends	Indiv controls	State controls	State time trends	Indiv controls	State controls	State time trends	Indiv controls	State controls	State time trends
		(-0.006 to 0.025)	(-0.006 to 0.020)		(-0.005 to 0.006)	(-0.006 to 0.006)		(-0.002 to 0.017)	(-0.001 to 0.018)		(-0.003 to 0.005)	(-0.005 to 0.002)
Poverty Rate		-0.013	0.000		-0.002	0.005*		-0.006	0.001		-0.005*	-0.005*
		(-0.031 to 0.005)	(-0.011 to 0.012)		(-0.007 to 0.004)	(-0.000 to 0.010)		(-0.017 to 0.005)	(-0.009 to 0.010)		(-0.009 to 0.000)	(-0.010 to 0.001)
Governor is Democrat		-0.032	-0.021		-0.001	-0.002		-0.024**	-0.022		-0.006	0.003
		(-0.084 to 0.021)	(-0.066 to 0.025)		(-0.026 to 0.023)	(-0.025 to 0.020)		(-0.048 to -0.001)	(-0.050 to 0.006)		(-0.021 to 0.008)	(-0.009 to 0.015)
State EITC Rate		0.790**	0.304		0.093	-0.037		0.590***	0.191		0.110	0.157**
		(0.126 to 1.454)	(-0.134 to 0.741)		(-0.187 to 0.372)	(-0.254 to 0.179)		(0.201 to 0.979)	(-0.101 to 0.483)		(-0.034 to 0.253)	(0.024 to 0.290)
TANF and SNAP 2 person benefit maximum		0.000	0.000		0.000	0.000		0.000	0.000		0.000	0.000
		(-0.001 to 0.001)	(-0.000 to 0.001)		(-0.000 to 0.000)	(-0.000 to 0.000)		(-0.001 to 0.001)	(-0.000 to 0.001)		(-0.000 to 0.000)	(-0.000 to 0.000)
Pregnant women Medicaid eligibility		0.084	-0.009		0.012	-0.121**		0.057	0.086*		0.013	0.031
		(-0.075 to 0.244)	(-0.173 to 0.155)		(-0.053 to 0.078)	(-0.222 to -0.020)		(-0.033 to 0.147)	(-0.012 to 0.183)		(-0.016 to 0.042)	(-0.024 to 0.086)
Parents Medicaid eligibility		0.044	0.013		0.015	0.013		0.022	-0.017		0.008	0.018***
		(-0.009 to 0.098)	(-0.030 to 0.055)		(-0.011 to 0.041)	(-0.013 to 0.039)		(-0.006 to 0.049)	(-0.038 to 0.004)		(-0.006 to 0.021)	(0.006 to 0.029)
Constant	2.194***	2.295***	2.296***	0.904***	0.935***	1.063***	1.032***	1.043***	1.070**	0.260***	0.323***	0.153
	(1.899 to 2.489)	(1.426 to 3.165)	(1.202 to 3.390)	(0.803 to 1.005)	(0.622 to 1.248)	(0.648 to 1.477)	(0.836 to 1.228)	(0.365 to 1.722)	(0.269 to 1.872)	(0.161 to 0.358)	(0.135 to 0.512)	(-0.086 to 0.392)
Observations	199,308	199,308	199,308	199,048	199,048	199,048	199,181	199,181	199,181	198,879	198,879	198,879
R-squared	0.066	0.066	0.068	0.061	0.061	0.062	0.040	0.041	0.042	0.030	0.030	0.032

NOTES HS=High School. AI/AN= American Indian/Alaska Native. PI=Pacific Islander. EITC=Earned Income Tax Credit. TANF=Temporary Assistance for Needy Families. SNAP=Supplemental Nutrition Assistance Program. 95% confidence intervals reflect clustered standard errors. *** p<0.01, ** p<0.05, * p<0.1

© 2023 Rokicki S et al. *JAMA Network Open.*

eTable 2. Two-Way Fixed Effect Estimates of $1 Increase in Minimum Wage on Individual Stressful Events in the 12 Months Before Delivery, 2004-2015

	**Model 1**	**Model 2**	**Model 3**	**N**	**Sample mean (SE)**
Got separated or divorced	-0.000	-0.002	-0.003	198,408	0.117
	(-0.007 to 0.007)	(-0.009 to 0.004)	(-0.009 to 0.003)		(0.001)
Argued with husband/partner more than usual	-0.006	-0.007*	-0.003	198,033	0.290
	(-0.014 to 0.002)	(-0.014 to 0.000)	(-0.014 to 0.008)		(0.002)
Husband/partner did not want pregnancy	-0.008**	-0.011***	-0.013***	198,001	0.100
	(-0.014 to -0.002)	(-0.017 to -0.004)	(-0.022 to -0.005)		(0.001)
Husband/partner lost his job	-0.004	-0.005	-0.009*	197,291	0.160
	(-0.012 to 0.005)	(-0.012 to 0.001)	(-0.017 to 0.000)		(0.002)
Respondent lost job even though wanted to keep working	-0.002	-0.004	-0.005	197,537	0.140
	(-0.010 to 0.005)	(-0.013 to 0.006)	(-0.016 to 0.005)		(0.001)
Had a lot of bills that couldn't pay	-0.001	-0.004	-0.008**	198,152	0.266
	(-0.008 to 0.006)	(-0.012 to 0.004)	(-0.015 to -0.001)		(0.002)
Moved to a new address	0.002	0.000	-0.009**	198,565	0.383
	(-0.007 to 0.011)	(-0.009 to 0.009)	(-0.016 to -0.002)		(0.002)
Husband/partner went to jail	-0.006*	-0.007*	-0.009**	198,189	0.069
	(-0.013 to 0.001)	(-0.016 to 0.001)	(-0.018 to -0.000)		(0.001)
Homeless	0.001	0.001	0.000	198,132	0.059
	(-0.005 to 0.008)	(-0.005 to 0.007)	(-0.006 to 0.007)		(0.001)
**Model specifications**					
State and year fixed effects	Yes	Yes	Yes		
Individual controls	Yes	Yes	Yes		
State time-varying controls	No	Yes	Yes		
State linear time trends	No	No	Yes		

Notes: SE=standard error of the mean. Sample mean and standard error are weighted. Individual controls include maternal age, race and ethnicity, marital status, parity, educational level, and month of child's birth. State time-varying controls include unemployment rate, gross state product, percentage of children uninsured, poverty rate, state Earned Income Tax Credit rate as a percentage of the federal rate, combined monthly maximum for Temporary Assistance for Needy Families and Supplemental Nutrition Assistance Program benefits for a 2-person family, whether the governor was affiliated with the Democratic party, and Medicaid eligibility level as a percentage of the federal poverty limit for pregnant women and for parents. 95% confidence intervals reflect clustered standard errors. *** p<0.01, ** p<0.05, * p<0.1

© 2023 Rokicki S et al. *JAMA Network Open.*

eTable 3. Incidence Rate Ratio Estimates Using Poisson Regression Models of $1 Increase in Minimum Wage on Stressful Life Events in the 12 Months Before Delivery, 2004-2015

	**Model 1 IRR**	**Model 2 IRR**	**Model 3 IRR**	**N**	**Sample mean (SE)**
Total stressful events	0.987	0.976*	0.958***	199,308	1.57
	(0.966 - 1.008)	(0.948 - 1.005)	(0.931 - 0.985)		(0.007)
Partner-related stressful events	0.978**	0.964**	0.956***	199,048	0.51
	(0.958 - 0.998)	(0.936 - 0.993)	(0.928 - 0.984)		(0.003)
Financial stressful events	0.995	0.986	0.964**	199,181	0.94
	(0.971 - 1.020)	(0.957 - 1.016)	(0.935 - 0.993)		(0.004)
Traumatic stressful events	0.973	0.959	0.936	198,879	0.13
	(0.891 - 1.062)	(0.871 - 1.056)	(0.842 - 1.040)		(0.001)
**Model specifications**					
Individual controls	Yes	Yes	Yes		
State and time fixed effects	Yes	Yes	Yes		
State time-varying controls	No	Yes	Yes		
State linear time trends	No	No	Yes		

Notes: IRR=incidence rate ratios. SE=standard error of the mean. Sample mean and standard error are weighted. Individual controls include maternal age, race and ethnicity, marital status, parity, educational level, and month of child's birth. State time-varying controls include unemployment rate, gross state product, percentage of children uninsured, poverty rate, state Earned Income Tax Credit rate as a percentage of the federal rate, combined monthly maximum for Temporary Assistance for Needy Families and Supplemental Nutrition Assistance Program benefits for a 2-person family, whether the governor was affiliated with the Democratic party, and Medicaid eligibility level as a percentage of the federal poverty limit for pregnant women and for parents. 95% confidence intervals reflect clustered standard errors. *** p<0.01, ** p<0.05, * p<0.1

© 2023 Rokicki S et al. *JAMA Network Open.*

eTable 4. Two-Way Fixed Effect Estimates of $1 Increase in Minimum Wage in the 12 Months Before Delivery on Total Stressful Life Events, Stratified by Characteristics, 2004-2015

	Total stressful life events	N	Sample mean (SE)
**Race and ethnicity**			
American Indian/Alaska Native	0.139 (-0.108 to 0.385)	10,436	1.730 (0.036)
Asian/Pacific Islander	0.020 (-0.224 to 0.264)	9,688	1.083 (0.026)
Hispanic	-0.125** (-0.242 to -0.009)	45,641	1.391 (0.013)
Black	-0.047 (-0.125 to 0.031)	39,028	1.764 (0.015)
White	-0.036* (-0.074 to 0.001)	88,038	1.625 (0.009)
**Marital status**			
Not married	-0.074*** (-0.125 to -0.023)	119,201	1.841 (0.009)
Married	-0.045*** (-0.078 to -0.012)	80,107	1.185 (0.009)
**Age**			
Under 30 years	-0.055*** (-0.092 to -0.017)	152,549	1.684 (0.008)
30 years or over	-0.073** (-0.139 to -0.008)	46,759	1.213 (0.012)

Notes: SE=standard error of the mean. Sample mean and standard error are weighted. All models adjusted for individual covariates, state and year fixed effects, and state linear time trends. Individual controls include maternal age, race and ethnicity, marital status, parity, educational level, and month of child's birth. State time-varying controls include unemployment rate, gross state product, percentage of children uninsured, poverty rate, state Earned Income Tax Credit rate as a percentage of the federal rate, combined monthly maximum for Temporary Assistance for Needy Families and Supplemental Nutrition Assistance Program benefits for a 2-person family, whether the governor was affiliated with the Democratic party, and Medicaid eligibility level as a percentage of the federal poverty limit for pregnant women and for parents. 95% confidence intervals reflect clustered standard errors. *** p<0.01, ** p<0.05, * p<0.1

© 2023 Rokicki S et al. *JAMA Network Open.*

eTable 5. Results of Robustness Checks and Falsification Tests

	Estimate on total stressful life events	N
**Panel A. Robustness checks**		
Restricted sample to states with <3 missing years (24 total states)	-0.061***	146,389
	(-0.104 to -0.019)	
Including phase 8 (study period 2004-2019)	-0.036**	240,168
	(-0.069 to -0.003)	
Alternative exposure: nominal MW wage	-0.075***	199,308
	(-0.119 to -0.031)	
Alternative exposure: real MW in year before delivery	-0.064***	199,308
	(-0.091 to -0.038)	
Main specification with Wild Cluster Bootstrap 95%CI	-0.060***	199,308
	(−0.102, −0.019)	
Exclude individuals with any missing data on stressors	-0.054***	193,124
	(-0.090, -0.018)	
**Panel B. Falsification tests**		
Among college educated sample	-0.007	248,790
	(-0.020 to 0.006)	
Among high school or less sample	-0.060***	199,308
	(-0.095 to -0.024)	
Future exposure: 1 year after delivery	0.026	199,308
	(-0.005 to 0.057)	
Future exposure: 2 years after delivery	0.001	199,308
	(-0.022 to 0.024)	
Future exposure: 4 years after delivery	-0.005	199,308
	(-0.043 to 0.033)	

NOTES MW=minimum wage. All models are adjusted for individual covariates, state and year fixed effects, and state linear time trends. Individual controls include maternal age, race and ethnicity, marital status, parity, educational level, and month of child's birth. State time-varying controls include unemployment rate, gross state product, percentage of children uninsured, poverty rate, state Earned Income Tax Credit rate as a percentage of the federal rate, combined monthly maximum for Temporary Assistance for Needy Families and Supplemental Nutrition Assistance Program benefits for a 2-person family, whether the governor was affiliated with the Democratic party, and Medicaid eligibility level as a percentage of the federal poverty limit for pregnant women and for parents. 95% confidence intervals reflect clustered standard errors. *** p<0.01, ** p<0.05, * p<0.1

© 2023 Rokicki S et al. *JAMA Network Open.*
