# Supplementary material for: Association of Increasing the Minimum Wage in the US With Experiences of Maternal Stressful Life Events
Source: JAMA Netw Open. 2023 Jul 18;6(7):e2324018. doi: 10.1001/jamanetworkopen.2023.24018 (PMC10354676; doi:10.1001/jamanetworkopen.2023.24018)
Supplement: Supplement 2. — Data Sharing Statement [file jamanetwopen-e2324018-s002.pdf]

Data Sharing Statement

Rokicki. Association of Increasing the Minimum Wage in the US With Experiences of Maternal Stressful Life Events. *JAMA Netw Open*. Published July 18, 2023.
doi:10.1001/jamanetworkopen.2023.24018

Data
Data available: No

Additional Information
Explanation for why data not available: PRAMS data is publicly available for researchers on https://www.cdc.gov/prams/prams-data/researchers.htm.
